# A mini-review: Bridging the gap between autism spectrum disorder and pain comorbidities

**DOI:** 10.1080/24740527.2020.1775486

**Published:** 2020-12-30

**Authors:** Chad O. Brown, Jarryll Uy, Karun K. Singh

**Affiliations:** a Department of Biochemistry and Biomedical Sciences, McMaster University, Hamilton, Ontario, Canada; b Stem Cell and Cancer Research Institute, McMaster University, Hamilton, Ontario, Canada; c Michael G. DeGroote Institute for Pain Research and Care, McMaster University, Hamilton, Ontario, Canada

**Keywords:** autism spectrum disorder, *SCN9A*, Na_v_1.7, chronic pain, sensory

## Abstract

**Background:**

Pain is a complex neurobiological response with a multitude of causes; however, patients with autism spectrum disorder (ASD) often report chronic pain with no known etiology. Recent research has been aimed toward identifying the causal mechanisms of pain in mouse and human models of ASD. In recent years, efforts have been made to better document and explore secondary phenotypes observed in ASD patients in the clinic. As new sequencing studies have become more powered with larger cohorts within ASD, specific genes and their variants are often left uncharacterized or validated. In this review we highlight ASD risk genes often presented with pain comorbidities.

**Aims:**

This mini-review bridges the gap between two fields of literature, neurodevelopmental disorders and pain research. We discuss the importance of the genetic landscape of ASD and its links to pain phenotypes.

**Results:**

Among the numerous genes implicated in ASD, few have been implicated with varying severities of pain comorbidity. Mutations in these genes, such as *SCN9A, SHANK3*, and *CNTNAP2*, lead to altered neuronal function that produce different responses to pain, shown in both mouse and human models.

**Conclusion:**

There is a necessity to use new technologies to advance the current understanding of ASD risk genes and their contributions to pain. Secondly, there is a need to power future ASD risk genes associated with pain with their own cohort, because a better understanding is needed of this subpopulation.

## RÉSUMÉ

**Contexte**: La douleur est une réponse neurobiologique complexe dont les causes sont multiples ; cependant, les patients atteints de troubles du spectre de l’autisme (TSA) rapportent souvent une douleur chronique sans étiologie connue. Des recherches récentes ont visé à identifier les mécanismes causaux de la douleur chez des modèles murins et humains.

Ces dernières années, des efforts ont été faits pour mieux documenter et étudier les phénotypes secondaires observés chez les patients atteints de TSA en clinique. Étant donné que les nouvelles études de séquençage sont devenues plus puissantes et se réalisent avec des cohortes plus importantes au sein des TSA, des gènes spécifiques et leurs variantes demeurent souvent non caractérisés ou validés. Dans cette revue, nous mettons en évidence les gènes de risque de TSA qui se présentent souvent avec des comorbidités douloureuses.

**Objectifs**: Cette mini-revue comble le fossé entre deux domaines de la littérature, les troubles neurodéveloppementaux et la recherche sur la douleur. Nous discutons de l’importance du paysage génétique des TSA et de ses liens avec les phénotypes de la douleur.

**Résultats**: Parmi les nombreux gènes impliqués dans les TSA, peu ont été impliqués avec divers de degrés de sévérité de la comorbidité de la douleur. Des mutations dans ces gènes, tels que SCN9A, SHANK3 et CNTNAP2, conduisent à une fonction neuronale altérée qui produit des réponses différentes à la douleur, que l’on retrouve à la fois chez les modèles murins et humains.

**Conclusion**: Il est nécessaire d’utiliser les nouvelles technologies pour faire progresser la compréhension actuelle des gènes de risque de TSA et leurs contributions à la douleur. Deuxièmement, il est nécessaire d’augmenter la puissance des futurs gènes de risque de TSA associés à la douleur avec leur propre cohorte, car une meilleure compréhension de cette sous-population est nécessaire.

## Introduction

Pain is often described as a complex neurobiological response to an unpleasant sensory or emotional experience associated with actual or potential tissue damage.^1^^,2^ In recent years, an increased focus has been devoted to pain research because many individuals will experience subtypes of pain, whether chronic, which can last several months, or acute, which typically has a shorter duration, over their lifetime. In 2011, chronic pain in Canada was estimated to have affected 6 million adults (~19% of the population).^3^ Approximately half of all reports documented that patients suffered from chronic pain for more than 10 years, with one-third of all cases reporting the intensity to be very severe. Unfortunately, epidemiological systematic reviews that aim to examine the prevalence of chronic pain have reported an alarming importance of age range within population statistics. This was highlighted by the frequency of chronic pain peaking at 45 to 65 years of age found in 17 epidemiological studies ending in the year 2000. Prior to this most recent Canadian chronic pain prevalence estimate, many researchers highlighted an age-dependent frequency increase in chronic pain by 33% to 39% at 55 years of age and older. Additionally, reports suggested that females may experience higher severity of chronic pain than males.^4–6^ In upcoming years these estimates will only increase as the baby boomer generation, the largest subset of the Canadian population determined by Statistics Canada (~29% in 2011),^7^ continues to age into the major Canadian cohorts that experience chronic pain. A recent study estimated a weighted annual incremental direct cost to manage chronic pain across Canada of US$7.2 billion, which does not include additional expenses paid by the individual or indirect costs; taking these into account, the approximate total would range from US$56 to US$60 billion per year.^8,9^

Individuals with multiple neurological conditions have been diagnosed with chronic pain, including those with spinal cord injury, stroke, multiple sclerosis, and Parkinson’s disease.^10–21^ The prevalence of pain in people with neurological conditions is estimated to be twice that of the general population at 15% to 19%.^22^ Within this subcategory, approximately 84% of patients with traumatic spinal cord damage report chronic pain. In addition, the overarching theme of altered neural connectivity, which may present as central and peripheral neuropathic pain (42%) or nociceptive musculoskeletal pain (71%), emphasizes the importance of investigating all neurological conditions and their association with chronic pain.^23,24^

Unfortunately, neurological conditions such as neurodevelopmental disorders have not garnered much attention within the field of pain. One of the major neurodevelopmental disorders that presents comorbidity with chronic pain is autism spectrum disorder (ASD). ASD is defined by the core characteristics impaired social interaction and communication and restricted and repetitive behaviors.^25^ ASD’s prevalence has continued to increase over the last 50 years: in 2018 the Canadian Public Health Agency determined the prevalence to be 1 in 66 children.^26^ A large number of studies have primarily focused on potential environmental risk factors as contributors for ASD, such as maternal diet, gut microbiota, prenatal exposures to toxins, and cesarean sections. Genetic and neuropathological studies have aimed to identify the causal mechanisms of ASD.^27–35^

In this review, we aim to provide an overview of the literature highlighting the importance of genetic studies in neurodevelopmental and pain research. One gene that has recently appeared in both bodies of literature without researchers merging their efforts to gain a better molecular and cellular understanding is the voltage-gated sodium channel, named *SCN9A*. We hope to bridge these fields toward a common goal of improving patient outcomes while also elucidating potential functional characterization approaches to provide targeted therapeutic approaches to patients with ASD and pain comorbidities.

## Genetics of Autism Spectrum Disorder

Among the determinants of ASD, genetics are largely known to play a key role in the disease pathophysiology. Previous monozygotic twin studies revealed a large genetic component of ASD, with heritability rates between 50% and 90%.^36^ Recently, a large population-based multinational study estimated the general heritability of ASD as approximately 80%.^37^ Genome-wide association studies were used initially to identify common genetic variants in the form of single nucleotide variations or copy number variations that might be enriched in populations with ASD versus their neurotypical counterparts. However, these studies have found few potential common variants and thus researchers have turned toward rare inherited variants (present in <1% of the population) and de novo mutations (present only in the affected child). Recent large-scale whole-genome and -exome sequencing studies have implicated numerous genes as associated with ASD.^38–41^ These studies have found multiple rare inherited and de novo single nucleotide variations and copy number variations that underlie the complex genetic landscape of ASD. Many of these genetic variants are categorized in the SFARI gene database, the foremost accepted database of ASD risk genes, using scores based on sequencing evidence and studies that look at the neurodevelopmental impact caused by disruptions of those genes.^42^. Based on the number of variants and their correlation to clinical and cellular phenotypes related to pain, the gene *SCN9A*, which encodes a voltage-gated sodium channel within the peripheral nervous system (PNS), has been designated as a category 2 gene in relation to ASD. This gene has started to garner attention due to its high penetrance and distinct pain phenotypes observed in patients with ASD with pain comorbidities. Thus, this gene has been the most studied ASD risk gene in relation to pain.

### SCN9A *the Pain Gene*

Pain response is a complex process that commonly involves transduction of nerve signals from the PNS to the autonomic and central nervous system (CNS), known as nociception. In addition to processing of nociceptive inputs, neuropathic pain conditions have their own unique origins of pain and processing. Neuropathic pain can be further divided into central or peripheral, where central is commonly a consequence of a previous disease or injury such as stroke, multiple sclerosis, and spinal cord injury that damages the CNS.^43^ More commonly observed is peripheral neuropathic pain, which originates from impairment to PNS pathways, including complex regional pain syndrome and postherpetic neuralgia.^43^ A key contributor to nociceptive neuron excitability that has emerged within recent years is the gene *SCN9A*, which encodes a voltage-gated sodium channel (Na_v_1.7).^44,45^ Na_v_1.7 is an ion channel subtype of the voltage-gated sodium family that is primarily expressed in the PNS, specifically in dorsal root ganglion, specialized sensory neurons known as nociceptive neurons, and other ganglion neurons.^46–48^ These neurons are crucial for signal transduction, which is important for pain. In addition to Na_v_1.7 expression in specialized sensory neurons, limited but emerging evidence has highlighted rare variant expression that decreases firing of specific inhibitory GABAergic neurons.^49,50^ Dorsal root ganglia are sites of clustering sensory neurons that propagate signals from the periphery, where nociceptors detect the threat of damaging stimuli, to the spinal cord. Nociceptors are highly modular, whether they take on more mechanosensitive functions responding to temperature, pressure, or chemosensitive to chemicals.^51^

Biophysical studies of Na_v_1.7 have implicated its function in action potential initiation and its regulation of subthreshold stimuli.^44,52–54^ Recent studies have highlighted efforts to sequence patients with pain phenotypes to determine a genetic etiology of pain syndromes such as erythromelalgia, paroxysmal extreme pain disorder, small fiber neuropathy, and channelopathy-associated congenital indifference to pain.^54–60^ A common observation was a convergence of unknown variants accumulating in *SCN9A*. This provided initial necessary observations, but further functional validation or phenotyping is needed to ensure that these variants are the cause of the pain syndromes observed in patients. Further evidence suggested that gain-of-function variants in *SCN9A* enhanced neuronal excitability, which was observed in erythromelalgia, paroxysmal extreme pain disorder, and small fiber neuropathy; loss-of-function variants recapitulated a hypoexcitable neuronal phenotype that exhibited as channelopathy-associated congenital indifference to pain. These experiments were primarily performed in HEK293 cells and, more recently, in human-derived sensory neurons with electrophysiology measuring both sodium currents and inactivation and activation properties of the currents. Many authors have highlighted the convergence of mutations in *SCN9A*, but a plethora of variants are still uncharacterized in patients with pain syndromes.

## The Autism Spectrum Disorder Risk Gene *SCN9A* and Pain Comorbidities

It is well known that ASD is a genetically heterogeneous disorder, where multiple genes contribute to a phenotype. Patients express the core characteristics of ASD but may also have secondary behavioral contributions or comorbidities to other diseases and disorders. Secondary or comorbid conditions that have been observed in patients with ASD include altered sensory sensitivity and pain.^61–67^ A recent investigation using data from 48,591 children without ASD, 1158 children with ASD, and 314 children with ASD and one other comorbid disorder found the prevalence of pain per group to be 8.2%, 15.6%, and 19.9%, respectively.^66^ This was on average double the prevalence observed in children without ASD. ASD is often conceptualized to involve disruption in neural signaling and, more specifically, caused by an imbalance in excitation or inhibition. Many sensory abnormalities found in the clinic align with the potential convergence at the cellular level of atypical neural connectivity. It has been shown that individuals with ASD exhibit an altered neural response to pain as demonstrated by functional magnetic resonance imaging.^68^ In addition, it has been reported that 95% of individuals with ASD have aberrant reactivity to sensory stimuli, including tactile stimuli, and varying severities of acute and chronic pain.^69^ Previous cases in the literature showed an overlap between ASD and chronic pain, particularly among adolescents.^6,70,72^ As previously mentioned, ASD has a strong genetic contribution, and advances of arrays and sequencing technologies have provided tools to elucidate both de novo single nucleotide variants and copy number variations and other genetic variants that contribute to approximately 10% to 40% of cases.^49,73–75^ Sodium channels in ASD research have gained significant traction, with *SCN2A*, a gene that encodes a voltage-gated sodium channel subtype 2 alpha protein (Na_v_1.2), becoming a front runner due to the sheer amount of de novo single nucleotide variants that occur in patients. The less studied *SCN9A* gene is important in the PNS and regulates sensory neuronal excitability. When looking outside of ASD research, *SCN9A* has been highly characterized in pain research. To date, there has only been one direct investigation that has linked all three components: *SCN9A*, ASD, and pain.^49^ In this study, researchers were able to identify two families from the National Institute of Mental Health repository and whole-exome sequence analyses of family members.^76^ *SCN9A* was chosen as one of the candidate genes based on the abundance of variants. One variant, Na_v_1.7^C1143F^, was validated and it was determined that there were changes in recovery of fast inactivation of the current.^49^ Furthermore, other biophysical neuronal properties such as input resistance and rheobase were altered, such that input resistance was lowered and rheobase increased, requiring a larger magnitude of current to evoke action potentials.^49^ A second variant was also further investigated, Na_v_1.7^M932L/V991L^, which has been previously implicated in neuropathic pain syndromes and was associated with partial deletion of pain perception.^60,77^ Biophysical properties of this variant remained unchanged but, similar to Na_v_1.7^C1143F^ cortical neurons, they fired fewer action potentials, indicating a reduced excitability phenotype. This finding highlighted the convoluted nature of predicting pain outcomes based on past clinically relevant information, without functional cellular validation. Previous investigations of Na_v_1.7^M932L/V991L^ suggest that this variant has the capacity to induce increased and decreased neuronal firing, raising the possibility that two different patients with the same mutation can exhibit varying pain phenotypes.^49,60,77^ Lastly, it is necessary to consider the contributions of the neuron subtype, the auxiliary subunits, and the distinction between Na_v_1.7 expression in CNS and PNS, because ASD risk genes have had an underappreciated role in the PNS surrounding pain phenotypes. In summary, the authors were able to functionally validate known variants in pain research, Na_v_1.7^C1143F^ and Na_v_1.7^M932L/V991L^, and continue to probe other unknown variant functions discovered from their sequencing results of patients with ASD.

Among the genes in the SFARI database, multiple genes have been identified as being implicated in pain phenotypes. One such gene is *SHANK*3, a gene mutated in Phelan-McDermid syndrome, where primary symptoms include ASD as well as blunted pain sensitivity.^78^ Furthermore, in a study evaluating 201 patients with Phelan-McDermid syndrome, it was found that nearly 80% demonstrated insensitivity to pain.^79^ Mice lacking one or both copies of *Shank3* have been shown to have an insensitivity to pain.^80^ Han et al.^80^ showed that deletion of *Shank3* in peripheral sensory neurons of mice led to a decrease in inflammatory pain sensitivity. It was further shown that the specific deletion of *Shank3* in sensory neurons recapitulated pain deficits in *Shank3* global knockout mice. This finding suggested that insensitivity to pain seen in patients with *SHANK3* mutations may be attributed not solely to dysfunction in the brain but within the PNS. Mutations in the mouse model of ASD harboring deletions in *Cntnap2* have been shown to display hypersensitivity to pain.^81^ Dawes et al.^81^ demonstrated that sensory neurons of *Cntnap2*^−/−^ mice had impaired K_v_1 channel function that produced unregulated neuronal excitability. They identified the key role of an ASD-associated gene in pain sensitivity and examined potential mechanisms by which it occurs. Additionally, Sener et al.^82^ examined expression of genes related to aggression and pain sensitivity in whole-blood samples of patients with ASD. They found altered expression of several genes such as *SCN9A* and *OPRM1*, which are genes related to pain as well as significantly higher expression of the aggression-related *TACR1* gene in patients with ASD.^82^ This study provided novel insight into potential biomarkers of the consequent behavioral phenotypes of individuals diagnosed with ASD. Understanding of the links between ASD and pain remains poor and thus highlights the need to further identify the underlying causal mechanisms for future therapeutic intervention.

## Concluding Remarks

It is evident that there is a wealth of knowledge within each of the respective fields of ASD and pain, but to increase the success of patient outcomes, more will need to be done to draw parallels between fields and to allow future experiments to emphasize this bridge. As both fields evolve, more human-relevant experiments will be needed. Most current neuroscience experiments use mouse models to investigate the human condition. As the field is starting to note, pathways, proteins, and overall cellular dynamics are not always recapitulated. To further highlight the relevance of *SCN9A* variants in clinically relevant settings, models such as induced pluripotent stem cells, which can be reprogrammed from healthy or patient somatic cells, can be used as a powerful tool.^83^ These induced pluripotent stem cells have been well characterized, with advancements in protocols to finely tune differentiation targeted toward cell and tissue types like cortical neurons, sensory neurons, cardiomyocytes, gastrointestinal cells, and more.^84–87^ Furthermore, these protocols allow for improved scalability for experiments that may help expedite the process of functionally characterizing specific variants in human-derived tissue. In tandem with new technologies such as microelectrode arrays, this may provide a functional platform that may be used in the future for screening patient mutations and allow for drug screens to occur on a large scale. Targeted patient therapeutics would thus be enhanced and allow for preliminary screening to occur within the clinic without compromisin patient safety (e.g., toxic symptoms or drug treatments that would produce no benefit).
